# Proteomics analysis reveals differentially activated pathways that operate in peanut gynophores at different developmental stages

**DOI:** 10.1186/s12870-015-0582-6

**Published:** 2015-08-04

**Authors:** Chuanzhi Zhao, Shuzhen Zhao, Lei Hou, Han Xia, Jiangshan Wang, Changsheng Li, Aiqin Li, Tingting Li, Xinyou Zhang, Xingjun Wang

**Affiliations:** Bio-Tech Research Center, Shandong Academy of Agricultural Sciences; Shandong Provincial Key Laboratory of Crop Genetic Improvement, Ecology and Physiology, Jinan, 250100 P.R. China; Henan Academy of Agricultural Sciences, Zhengzhou, 450002 P.R. China

## Abstract

**Background:**

Cultivated peanut (*Arachis hypogaea*. L) is one of the most important oil crops in the world. After flowering, the peanut plant forms aboveground pegs (gynophores) that penetrate the soil, giving rise to underground pods. This means of reproduction, referred to as geocarpy, distinguishes peanuts from most other plants. The molecular mechanism underlying geocarpic pod development in peanut is poorly understood.

**Results:**

To gain insight into the mechanism of geocarpy, we extracted proteins from aerial gynophores, subterranean unswollen gynophores, and gynophores that had just started to swell into pods. We analyzed the protein profiles in each of these samples by combining 1 DE with nanoLC-MS/MS approaches. In total, 2766, 2518, and 2280 proteins were identified from the three samples, respectively. An integrated analysis of proteome and transcriptome data revealed specifically or differentially expressed genes in the different developmental stages at both the mRNA and protein levels. A total of 69 proteins involved in the gravity response, light and mechanical stimulus, hormone biosynthesis, and transport were identified as being involved in geocarpy. Furthermore, we identified 91 genes that were specifically or abundantly expressed in aerial gynophores, including pectin methylesterase and expansin, which were presumed to promote the elongation of aerial gynophores. In addition, we identified 35 proteins involved in metabolism, defense, hormone biosynthesis and signal transduction, nitrogen fixation, and transport that accumulated in subterranean unswellen gynophores. Furthermore, 26 specific or highly abundant proteins related to fatty acid metabolism, starch synthesis, and lignin synthesis were identified in the early swelling pods.

**Conclusions:**

We identified thousands of proteins in the aerial gynophores, subterranean gynophores, and early swelling pods of peanut. This study provides the basis for examining the molecular mechanisms underlying peanut geocarpy pod development.

**Electronic supplementary material:**

The online version of this article (doi:10.1186/s12870-015-0582-6) contains supplementary material, which is available to authorized users.

## Background

Cultivated peanut (*Arachis hypogaea*. L) is one of the most important oil crops in the world. Peanut seeds are composed of approximately 50 % high quality edible oil and about 30 % vegetable protein. Geocarpic pod development, or the development of underground pods, distinguishes peanuts from *Glycine max* (soybean), *Pisum sativa* (pea), and many other legume species. Geocarpic development enables the underground pods to absorb soil nutrients and protects the fruit from damage by ground animals and extreme weather. However, seeds that developed underground are more vulnerable to be infected by soilborne pathogens, for example *Aspergillus flavus*, and subsequent contamination with aflatoxins [1].

After fertilization, the gynophore forms, and elongates in a positive geotropic manner towards the ground. The gynophore elongates continuously and the ovary, which is located at the tip of the gynophore, does not swell until the gynophore has penetrated the soil. Previous studies suggested that light, mechanical stimuli, and hormones are all key factors in initiating pod swelling [2]. Before and after gynophore soil penetration, the phytochrome protein content changes significantly [3, 4], and light is considered to be the major factor preventing peanut pod development [5]. After gynophore soil penetration, the endogenous ethylene and IAA content change. Compared with aerial green gynophores, the underground so-called white gynophores release twice the amount of ethylene. However, the IAA content is high in the aerial gynophores and low in the swelling pod stage [6]. It is assumed that these physiological changes restart the development of the proembryo, and subsequently initiate the process of pod development and inhibit elongation of the gynophores [7]. In addition to the above three factors, gravity, calcium, and pH might also be important for the growth of the pod [2, 8].

Recently, several efforts were made to investigate the mechanisms of pod formation and development at the molecular level. Chen et al. (2013) analyzed the gene expression profiles of the peanut gynophore and young pod using 454 sequencing. They identified two senescence-associated genes that were significantly up-regulated in the aerial gynophores and suggested that these genes may contribute to embryo abortion in aerial gynophores [9]. In our recent study, we constructed a transcriptome library and analyzed the overall gene expression profile in the aerial-grown green gynophore, dark-grown white gynophore, and dark-grown gynophore with small pods (2–3 mm in length) using a HiSeq2000 system. We identified a global change in gene expression before and after the initiation of pod growth [10]. Furthermore, Zhu et al. (2013) performed a comparative proteomic analysis between the peanut aerial gynophore and subterranean pods using two-dimensional electrophoresis (2-DE) combined with mass spectrometry, and identified several candidate proteins related to pod swelling [11]. Using 2-DE proteomic analysis, another study identified 27 proteins that are differentially expressed in aerial gynophores, subterranean gynophores, and gynophores subjected to various treatments [12]. These results provided valuable information about proteome alteration before and after peanut pod swelling. However, due to the limited number of proteins identified, these studies fail to provide a comprehensive interpretation of the molecular mechanism underlying peanut geocarpy. To gain insight into the molecular networks underlying this unique and complex developmental process, more comprehensive proteomic profiling studies are required.

**Fig. 1:**
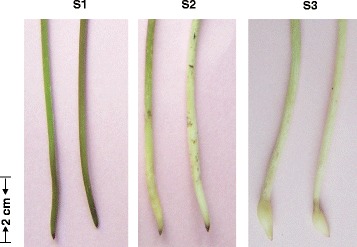
Gynophores of three different developmental stages used for protein extraction. Two representative examples each of S1, S2, and S3 gynophores are shown

Here, we report the identification of a reference proteome comprising 2766, 2518, and 2280 unique proteins from stage 1 (S1) gynophores (i.e., aerial gynophores that are green or purple and 3–5 cm long), stage 2 (S2) gynophores (i.e., gynophores that have been buried in the soil for about 3 days; the gynophores are white and ovary enlargement is not apparent), and stage 3 (S3) gynophores (dark-grown gynophores with 2–3 mm long pods) (Fig. 1). Among the identified proteins, those with ontologies related to gravity response, light and mechanical stimulus, hormone synthesis and transportation, and calcium-related proteins were prominent. This study lays the foundation for understanding the mechanisms of peanut gynophore and pod development at the protein level.

## Results

### Protein identification and statistical analysis

**Fig. 2:**
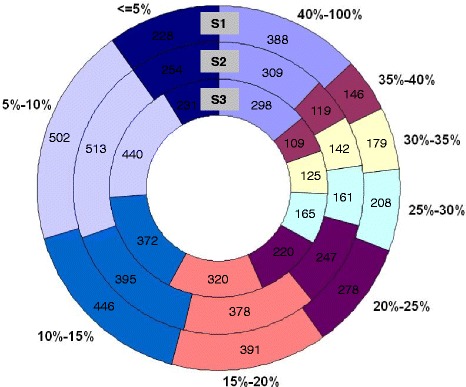
Distribution of the coverage of protein sequences mapped by peptides. According to the percentage of proteins covered by peptides, the identified proteins were divided into nine categories, 40-100 %, 35-40 %, 30-35 %, 25-30 %, 20-25 %, 15-20 %, 10-15 %, 5-10 %, and < =5 % of coverage. Numbers in the diagram indicate the quantity of proteins within the range of coverage. For example, there are 388, 309, and 298 proteins with a peptide coverage between 40 % and 100 % in S1, S2, and S3, respectively

**Fig. 3:**
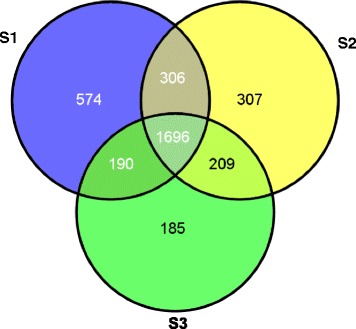
Venn diagram showing the number of proteins detected in S1, S2, and S3 samples. Numbers shared by two circles represent proteins shared by the two categories. The number shared by all three circles indicates the number of proteins found in all three samples

To acquire a comprehensive proteome profile of pods in the early stages of development, we combined 1 DE with nanoLC-MS/MS (liquid chromatography-tandem mass spectrometry). In total, 14,337 peptides were analyzed, resulting in the identification of 2766, 2518, and 2280 unique proteins in S1, S2, and S3 gynophores, respectively. The relative molecular mass of these proteins ranged from 3.5-136.8 kDa, and the false discovery rates (FDRs) were 1.09, 1.19, and 1.6 % in S1, S2, and S3 gynophores, respectively (Table 1). The distribution of coverage of protein sequences mapped by peptides is shown in Fig. 2. We identified 574, 307, and 185 specific proteins in S1, S2, and S3 gynophores, respectively (Fig. 3, Additional file 1: Table S1, Additional file 2: Table S2 and Additional file 3: Table S3). Three samples shared 1696 common proteins (Fig. 3, Additional file 4: Table S4). The best hit ID, sequence coverage, molecular weight, corresponding protein description, and sequences of all the identified proteins are summarized in Additional file 1: Table S1, Additional file 2: Table S2, Additional file 3: Table S3 and Additional file 4: Table S4. The number of peptides and number of spectra for the identified protein groups are also indicated in Additional file 1: Table S1, Additional file 2: Table S2, Additional file 3: Table S3 and Additional file 4: Table S4. The identified protein was defined as a reproducible protein if it was matched with at least two peptides. In total, 1049 proteins were detected with at least two matching peptides per protein. Sixteen of these proteins, such as ATP synthase, heat shock proteins, and WD-40 repeat family proteins, were detected at least 10 times, suggesting that they are relatively abundant. These relatively high abundant proteins may be involved in essential functions during gynophore and pod development. Many proteins were detected by a single peptide, suggesting that their abundance is low in the three samples (Additional file 1: Table S1, Additional file 2: Table S2, Additional file 3: Table S3 and Additional file 4: Table S4).

**Table 1:** Total proteins identified from gynophores of three developmental stages

Sample^(a)^	Total spectra^(b)^	Identified spectra^(c)^	Identified peptides^(d)^	Identified proteins^(e)^	FDR (%)^(f)^
S1	112003	31173	5435	2766	1.09
S2	105218	25668	4608	2518	1.19
S3	114294	24405	4294	2280	1.16
Total	331515	81246	14337	7564	-

^a^Three samples used in this study. S1: Aerial downward-growing gynophores; S2: Subterranean gynophores that had been buried in the soil for about three days; S3: Gynophores with early swelling pods that are buried in the soil. ^b^The total number of spectra. ^c^The number of spectra with an ion score above the threshold. ^d^The number of peptides matching the spectrum after repetitions of peptides had been removed. ^e^The number of proteins that the identified peptide matched, these proteins have at least one unique peptide. ^f^False discovery rate, which was set to less than 5 %

### Protein classification and functional annotation

**Fig. 4:**
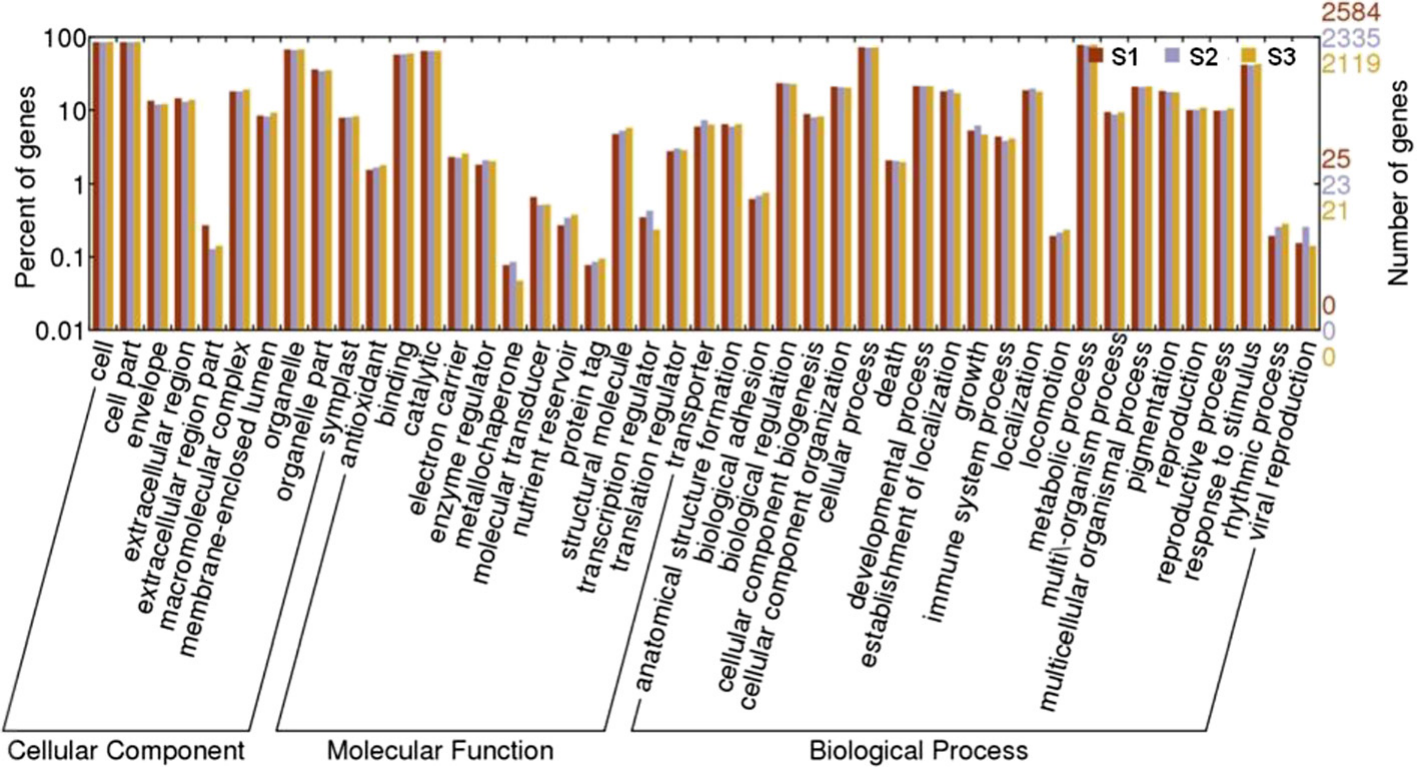
Histogram presentation of Gene Ontology classification. The identified proteins are grouped into three main categories: biological process, cellular component, and molecular function. The right y-axis indicates the number of genes in a category. The left y-axis indicates the percentage of a specific category of genes in that main category

To investigate the diverse functions of proteins obtained from the three samples, we predicted the biological functions of the identified proteins using Gene Ontology (GO) analysis (http://www.geneontology.org). Based on sequence homology, 2584, 2335, and 2119 proteins of S1, S2, and S3 gynophore samples were categorized into 45 functional groups (Fig. 4). In some cases, a particular protein could be classified into more than one category, suggesting that the protein has roles in multiple biological processes. Proteins involved in cell, cell part, metabolic process, cellular process, organelle, binding, catalytic activity, and response to stimulus were highly represented in all three samples.

**Fig. 5:**
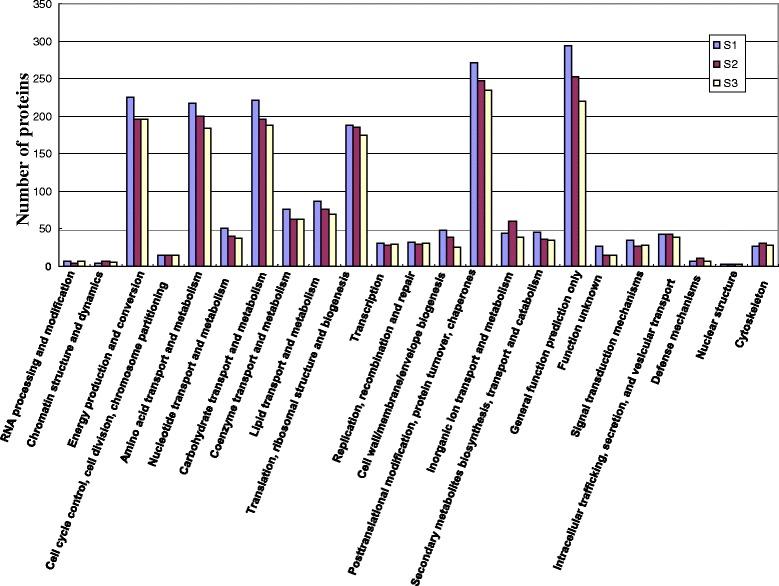
COG function classification of identified proteins. The identified proteins were classified into 23 categories, such as posttranslational modification, energy production and conversion, carbohydrate transport and metabolism, translation, and ribosomal structure. The y-axis indicates the number of genes in each category. The x-axis indicates the categories of proteins

To further evaluate the identified proteins, we performed Cluster of Orthologous Groups (COG) classification analysis. Among the 23 COG categories, the category of general function prediction only contained 271, 247, and 234 proteins in S1, S2, and S3, respectively, and represented the largest group, followed by the category of posttranslational modification, energy production and conversion, carbohydrate transport and metabolism, translation, ribosomal structure, and biogenesis. Nuclear structure, chromatin structure and dynamics, and RNA processing and modification represented the smallest groups, each containing only a few proteins (Fig. 5).

To identify the biological pathways that are active in S1, S2, and S3 gynophores, we mapped the identified proteins to the reference canonical pathways in the Kyoto Encyclopedia of Genes and Genomes (KEGG) database [13]. In total, 290, 282, and 278 KEGG pathways were represented by proteins identified in S1, S2, and S3 gynophores, respectively, and these pathways included metabolic pathways, plant-pathogen interaction pathways, plant hormone signal transduction pathways, and fatty acid biosynthesis pathways (Additional file 5: Table S5). These annotations provide a valuable resource for investigating specific processes and pathways associated with peanut gynophore and pod development.

### Proteins associated with peanut pod development

Previous studies suggested that the geocarpic development of peanut pods is regulated by several factors, such as light, mechanical stimuli, hormones, and gravity. To identify homologues of proteins involved in peanut pod development, we performed a BLASTP search against the NCBI Nr database (E-value < =10^−5^). In total, we identified 69 proteins with functions closely related to the above-mentioned key factors that regulate early pod development in peanut (Table 2). Thirteen of these proteins were associated with hormone metabolism and transport. Among these proteins, ten were involved in auxin biosynthesis, regulation, and transportation, such as auxin conjugate hydrolase, auxin-induced proteins, auxin-binding proteins, and auxin efflux carrier. One protein was involved in ethylene biosynthesis, one was related to brassinosteroid biosynthesis, and one was related to gibberellin regulation. Moreover, we identified 26 proteins related to gravity stimulation in the peanut proteome dataset, half of which were heat shock proteins (Table 2). Twenty-six proteins involved in light and mechanical stimuli were identified, and half of these (13) were annotated as peroxidases. In addition, five calcium-related proteins, namely calmodulin 5, calcium-binding protein, endoplasmic reticulum-type calcium-transporting ATPase 4, calcium-dependent protein serine/threonine kinase, and calcium-binding EF hand family protein, were identified (Table 2).

**Table 2:** The identified proteins associated with early pod development in peanut

Function category^(a)^	Protein annotation^(b)^	Accession no.^(c)^	Protein mass (Da)^(d)^	Number of unique spectra^(e)^			Expression level (RPKM)^(f)^		
				S1	S2	S3	S1	S2	S3
Hormone synthesis and transport									
	ethylene-forming-enzyme-like dioxygenase	Unigene1601	7294.74	1	1	1	40.79	39.57	29.88
	auxin-induced protein PCNT115	Unigene35781	7591	10	2	4	-	-	-
	auxin conjugate hydrolase	Unigene62418	15754.03	2	2	5	31.34	24.86	16.44
	auxin conjugate hydrolase	Unigene66297	18462.26	3	4	3	14.39	57.75	14.66
	auxin-induced protein	Unigene33790	7048.87	6	3	6	-	-	-
	auxin-induced protein	Unigene29960	6562.45	2	1	1	-	-	-
	auxin-induced-related/indole-3-acetic acid induced-related-like	Unigene70406	25322.62	2	0	0	9.56	15.51	13.71
	auxin binding protein 1	Unigene66527	21392.72	1	0	0	-	-	-
	auxin conjugate hydrolase	Unigene28944	4178.08	1	0	0	37.82	34.91	19.87
	auxin efflux carrier	Unigene68037	23205.64	1	0	0	42.66	13.21	5.53
	auxin efflux carrier family protein	Unigene72169	49455.3	1	0	0	-	-	-
	Gibberellin-regulated protein GA	Unigene10067	11170.06	38	29	43	-	-	-
	brassinosteroid biosynthetic protein LKB	Unigene62672	17576.9	2	11	5	311.10	206.03	49.91
Gravity stimulation									
	ABC transporter ABCE.2	Unigene72502	69066.7	39	23	26	84.41	82.15	87.00
	ABC transporter family protein	Unigene21711	5989.96	8	6	1	-	-	-
	white-brown-complex ABC transporter family	Unigene18576	49021.47	2	4	3	25.91	37.09	16.94
	ABC transporter family protein	Unigene70598	35095.19	2	2	1	75.21	68.28	80.76
	multidrug/pheromone exporter MDR family ABC transporter family	Unigene62432	16133.34	1	0	0	62.54	31.35	10.29
	PDR-type ABC transporter 2	Unigene71859	44479.44	2	0	0	48.56	188.56	91.96
	ABC transporter family protein	Unigene69124	18228.19	2	0	0	64.31	47.34	35.95
	ATNAP6 (NON-INTRINSIC ABC PROTEIN 6); protein binding/transporter	Unigene71274	36264.21	1	0	0	-	-	-
	ABC transporter family protein	Unigene65236	19643.19	1	0	0	-	-	-
	DnaJ protein	Unigene63710	16714.22	5	5	9	-	-	-
	DNAJ heat shock N-terminal domain-containing protein	Unigene69504	29786.75	2	0	0	22.64	11.06	12.10
	DNAJ heat shock family protein	Unigene61156	13833.69	1	0	0	-	-	-
	microtubule-associated protein MAP65-1a	Unigene71866	45884.12	0	0	1	94.44	122.33	209.65
	microtubule-associated protein	Unigene71117	33626.74	0	0	1	-	-	-
	heat shock protein 70-3	Unigene69094	27928.34	8	8	5	256.72	227.57	51.51
	heat shock protein 70	Unigene65006	18141.33	13	19	13	149.25	184.87	64.85
	heat shock 70 kDa protein mitochondrial	Unigene77	44585.2	73	35	49	97.49	75.41	38.98
	heat shock protein 70	Unigene64820	18289.48	20	18	29	-	-	-
	Heat shock 70 kDa protein mitochondrial	Unigene22321	5492.85	22	20	24	-	-	-
	heat shock protein 90	Unigene71426	41188.91	20	15	14	396.06	249.15	117.10
	heat shock protein 90	Unigene70349	34936.08	41	34	17	270.06	242.44	89.72
	heat shock protein 60	Unigene19291	13460.84	20	28	20	-	-	-
	heat shock 70 kDa protein mitochondrial	Unigene69299	18064.06	15	7	8	138.07	147.68	63.71
	heat shock protein 83	Unigene69604	31674.36	4	1	13	3.26	10.30	23.60
	heat shock protein binding protein	Unigene72153	46912.47	3	6	4	100.77	96.78	101.63
	peroxisomal small heat shock protein ACD31.2	Unigene55006	7984.84	6	9	13	-	-	-
Light and mechanical stimulus									
	vacuolar H + −ATPase B subunit	Unigene58237	12477.4	63	66	70	-	-	-
	vacuolar H + −ATPase B subunit	Unigene70168	23795.16	32	21	22	-	-	-
	V-H(+)-ATPase subunit A	Unigene57183	12256.11	63	54	51	-	-	-
	vacuolar proton-ATPase subunit-like protein	Unigene72042	53725.55	23	5	3	114.45	79.93	63.00
	vacuolar H + −ATPase A1 subunit isoform	Unigene22140	5891.07	26	10	23	-	-	-
	vacuolar (H+)-ATPase G subunit	Unigene66176	12282.34	6	4	3	-	-	-
	plasma membrane H + −ATPase	Unigene66682	10032.22	2	14	5	70.23	61.12	46.70
	autoinhibited H+ ATPase	Unigene71289	31534.9	3	2	2	96.02	100.12	66.26
	plasma membrane H+ ATPase	Unigene18749	33429.66	3	0	0	67.84	54.27	67.79
	plasma membrane Ca2 + −ATPase	Unigene58047	12779.62	1	0	0	28.12	10.97	10.64
	autoinhibited calcium ATPase	Unigene18942	20451.6	1	0	0	11.23	11.63	30.39
	plasma membrane H + −ATPase	Unigene59696	13366.89	2	0	0	87.35	99.89	62.04
	peroxidase	Unigene54700	11344.53	77	51	54	162.21	201.85	140.32
	peroxidase	Unigene57343	11907.84	66	72	94	-	-	-
	cytosolic ascorbate peroxidase	Unigene49105	9691.83	53	43	54	-	-	-
	peroxidase	Unigene70700	29953.04	43	52	31	291.66	397.48	55.60
	extensin peroxidase	Unigene46836	8747.6	31	31	34	-	-	-
	peroxidase 17	Unigene72081	36015.01	4	15	75	5.25	29.97	54.32
	glutathione peroxidase	Unigene63014	13139.73	10	7	12	-	-	-
	peroxidase	Unigene71860	35645.07	7	2	2	174.21	111.26	59.73
	peroxidase 30	Unigene28971	6729.43	3	1	5	348.59	269.03	79.47
	peroxidase precursor	Unigene48043	9252.78	3	12	6	49.16	38.76	5.94
	peroxidase 1 precursor	Unigene61708	14791.6	5	6	5	-	-	-
	peroxidase 1 precursor	Unigene42563	8357.1	7	2	8	-	-	-
	peroxidase	Unigene26422	6290.16	1	3	4	-	-	-
Calcium related									
	CAM5 (calmodulin 5); calcium ion binding	Unigene60884	6420.92	20	20	22	-	-	-
	calcium-binding protein	Unigene15783	6641.1	4	4	4	-	-	-
	endoplasmic reticulum-type calcium-transporting ATPase 4	Unigene71807	46699.82	4	7	5	80.84	75.27	72.45
	OST1 (open stomata 1); calcium-dependent protein serine/threonine kinase	Unigene3574	19012.44	2	1	1	-	-	-
	calcium-binding EF hand family protein	Unigene16448	44795.08	4	4	4	35.58	32.99	18.99

^a^Proteins associated with peanut pod development were classified into four categories: hormone synthesis and transport, gravity stimulation, light and mechanical stimulus, and calcium-related proteins. ^b^The function of these proteins was annotated using the Nr database. ^c^The corresponding accession numbers in the transcriptome database [10]. ^d^The molecular mass of proteins (Dalton). ^e^The number of unique spectra detected in each samples. ^f^The gene expression level corresponding to each protein

### Comparative analysis of the proteome and transcriptome of peanut

In our previous study, we performed a transcriptome analysis of peanut gynophores and pods (including S1, S2, and S3 samples), and generated a global gene expression profile that provided valuable information about the changes in gene expression that occur in S1, S2, and S3 gynophores and during pod development [10]. The gene expression level was calculated by RPKM (reads per kb per million reads). To evaluate the post-transcriptional regulation of gene expression during these three stages of gynophore development, we compared the protein identification results with the gene expression data. The correlation between identified proteins and mRNA expression is shown in Table 2. A total of 38 of the 69 identified key proteins were detected in the digital gene expression profiling results, including key proteins in hormone synthesis, response to gravity, light, mechanical stimulus, and calcium. Interestingly, the corresponding gene transcription levels of some proteins that were found to be highly abundant at the protein level (i.e., proteins that were detected by multiple peptides) were very low or undetected. Examples of these include gibberellin-regulated protein (Unigene10067), heat shock protein 70 (Unigene71426), heat shock protein 60 (Unigene19291), vacuolar H^+^-ATPase B subunit (Unigene58237, Unigene70168), and peroxidase (Unigene57343). By contrast, some highly expressed transcripts were not detected in the peanut proteome dataset. For example, the PDR-type ABC transporter 2 transcript (Unigene71859) exhibited higher expression levels in S2 (RPKM 188.56) and S3 (RPKM 91.96) gynophores than in S1 gynophores (RPKM 48.56). However, PDR-type ABC transporter 2 protein was only detected in S1 gynophores, suggesting the occurrence of post-transcriptional regulation of the corresponding gene.

## Discussion

Proteomic technology is a useful approach for identifying proteins in plants [14–18]. Recently, a number of proteins that are differentially expressed between peanut aerial gynophores, subterranean gynophores, and early swelling pods, were identified using two-dimensional electrophoresis combined with mass spectrometry [11, 12]. In the current study, we combined 1 DE with nanoLC MS/MS shotgun proteomic analysis to provide a comprehensive overview of the proteome profile during peanut gynophores and early pod development. Compared with traditional approaches, fractionation prior to LC − MS/MS analysis significantly increased the number of identified proteins and individual protein coverage [19]. We successfully identified 2766, 2518, and 2280 proteins in peanut S1, S2, and S3 gynophores, including 69 proteins related to the gravity response, light and mechanical stimuli, and hormone biosynthesis and transport, which are all considered to be important processes during peanut gynophore elongation and early pod development.

In contrast to other aboveground organs of peanut, the aerial gynophores grow downwards to the ground. Proteins that play positive roles in geotropic growth could thus be involved in gynophore development. It was reported that the ABC transporters, heat shock proteins, microtubules, and microtubule-associated proteins play important roles in the plant’s response to gravity stimulation [20, 21]. We identified 26 proteins related to gravity stimulation in the peanut proteome dataset, half of which were heat shock proteins (Table 2). Light and mechanical stimuli are the key factors affecting peanut pod development [2–5, 7]. In total, we identified 26 proteins involved in light and mechanical stimuli in this study, 13 of which were annotated as peroxidases. In addition, we identified five calcium-related proteins, i.e., calmodulin 5, calcium-binding protein, endoplasmic reticulum-type calcium-transporting ATPase 4, calcium-dependent protein serine/threonine kinase, and calcium-binding EF hand family protein (Table 2).

After soil penetration, the gynophores continue to elongate for a short period of time in the darkness. However, S2 gynophores exhibit morphological and physiological differences from S1 gynophores, indicating that the expression levels of some proteins were changed. Here, we detected 574 proteins that were specifically expressed in S1 gynophores. By comparing our proteomic data with our previously published RNA-seq dataset [10], we identified 91 proteins whose transcription levels were high or specifically expressed in S1 gynophores (Additional file 6: Table S6). For other proteins identified, the corresponding gene expression data were not in agreement with the protein accumulation patterns. We classified these 91 proteins into 11 groups according to their function. Among these specific proteins in S1 gynophores, 11 proteins were annotated as cell structure-related proteins, including four members of the pectin methylesterase family, two proteins involved in polygalacturonase, one expansin, one polygalacturonase-inhibiting protein, and two villins. Pectin methylesterase plays important roles in cell wall extension during pollen germination, pollen tube growth, and stem elongation [22]. Expansins are involved in cell enlargement and can cause irreversible extension of the cell wall [23], and have been linked to auxin action [24]. We suggest that the accumulation of pectin methylesterase and expansins promotes the elongation of S1 gynophores. By comparing our proteomic data with our previously published RNA-seq dataset, we also found some other interesting proteins in S1 gynophores (Additional file 6: Table S6). For example, GDSL-motif lipase, which is associated with ethylene signaling proteins in Arabidopsis [25], was identified only in S1 gynophores. At the transcription level, the GDSL-motif lipase unigene 69514 was strongly expressed in S1 gynophores (466.64 RPKM), while its expression levels were much lower in S2 (4.57 RPKM) and S3 (0.18 RPKM) gynophores.

We identified 307 proteins that were specifically or strongly expressed in S2 gynophores (Additional file 2: Table S2). The protein accumulation pattern of 35 of these proteins was in agreement with their transcription level in the S2 gynophore (Additional file 7: Table S7). However, the accumulation trend of other proteins was not in agreement with their transcripts. Function annotation and classification results showed that one-third of the proteins whose protein accumulation patterns agrees with their transcripts were enzymes involved in metabolism and secondary metabolism, such as anthocyanin-O-acyltransferase, caffeic acid O-methyltransferase, and phytase. In addition, three disease and defense-related proteins were identified, i.e., class III peroxidase, peroxidase 1 precursor, and peroxidase-like protein. Peroxidases are a family of isozymes found in plants, and their functions include removal of H_2_O_2_, biosynthesis and degradation of lignin in cell walls, auxin catabolism, defense responses to wounding, and defense against pathogen or insect attack [26]. We suggest that high levels of peroxidase accumulation play essential roles in underground gynophore development. Moreover, four transport-related proteins, namely amino acid/polyamine transporter II, MATE efflux family protein, PDR-type ABC transporter 2, and sugar transporter ERD6-like 6, were specifically identified in S2 gynophores.

Compared with the RNA-seq dataset [10], we found that 26 proteins of S3 were highly or specifically expressed both at the protein and mRNA level (Additional file 8: Table S8). Seven of these were enzymes involved in metabolism and secondary metabolism, including cinnamate 4-hydroxylase (C4H). C4H is a key catalytic enzyme in the synthesis of 4-hydroxycinnamate, a component in the lignin and anthocyanin biosynthesis pathways. In addition, fatty acids and starch synthesis-related proteins, such as lipoxygenase, FAD-binding domain-containing protein and starch branching enzyme I, were found in S3 gynophores and pods, suggesting that dry matter accumulation and seed development have started at this stage. Five cell growth or division and cell structure proteins were identified in early swelling pods. When the pod starts to swell, gynophore elongation ceases. The high levels of actin and microtubule-associated protein accumulation suggest that the cytoskeleton undergoes drastic change in S3 gynophores. The identification of specific and highly abundant proteins in S2 and S3 gynophores provided valuable information that can be used to advance our understanding of the molecular mechanism underlying geocarpic pod development.

## Conclusions

**Fig. 6:**
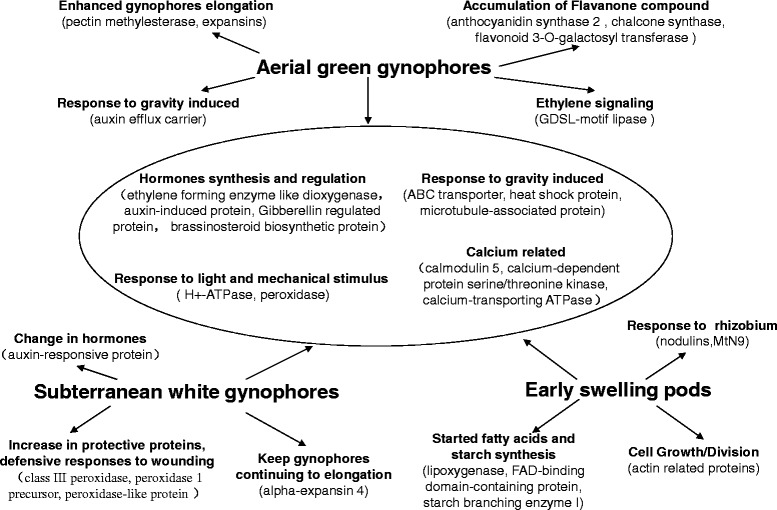
Identified proteins and their suggested function in peanut gynophores and during early pod development. Pectin methylesterases and expansins induced the aerial gynophores to grow and elongate. The ABC transporter, heat shock protein, microtubule-associated protein, and auxin efflux carrier controlled the direction of gynophore growth. After soil penetration, the new environment and soil friction induced an increase of protective proteins, such as class III peroxidase, peroxidase 1 precursor, and peroxidase-like protein. Furthermore, alpha-expansin 4 might prompt the gynophores to continue to elongate until the gynophores started to swell. After young pods formed, enzymes related to fatty acid metabolism and starch synthesis, such as lipoxygenase, FAD-binding domain-containing protein, and starch branching enzyme I, were activated. Meanwhile, the expression of actin-related proteins may reflect the pod development

Our integrated analysis of proteome and transcriptome data provided useful information about the mechanism controlling gynophore and pod development (Fig. 6). During aerial growth, the accumulation of pectin methylesterase and expansins promoted the growth and elongation of gynophores. The ABC transporter, heat shock protein, microtubule-associated protein, and auxin efflux carrier identified in S1 gynophores could play important roles in controlling the growth direction of gynophores. After soil penetration, the new environment and soil friction induced an increase in the levels of protective proteins, such as class III peroxidase, peroxidase 1 precursor, and peroxidase-like protein. Alpha-expansin 4, which might prompt gynophores to continue to elongate for a short period after gynophore soil penetration, was detected in the S2 sample. Once the young pods formed in S3 gynophores, the levels of enzymes related to fatty acid metabolism and starch biosynthesis increased, such as lipoxygenase, FAD-binding domain-containing protein, and starch branching enzyme I. Furthermore, actin-related proteins accumulated during early pod development. Interestingly, we observed that the nodulin gene and the corresponding protein were both strongly expressed during early pod initiation, suggesting that nodulation and early pod development might share similar mechanisms. Our proteome profiling results presented here provide useful insight into the mechanisms controlling peanut pod development.

## Methods

### Plant materials

Plants of “Luhua 14”, a widespread cultivar in Northern China, were sown in early May and harvested in early October in the experimental farm of Shandong Academy of Agricultural Sciences. The S1, S2, and S3 gynophores were collected from the farm-grown plants (Fig. 1). The tip-most 1 cm was excised from the S1, S2, and S3 gynophores and immediately frozen in liquid nitrogen for protein extraction.

### Protein extraction

Protein extraction was carried out according to the following method. Briefly, samples were ground to powder in liquid nitrogen, and then suspended in extraction solution (7 M urea, 2 M thiourea, 4 % CHAPS, 40 mM Tris–HCl (pH 8.5), 1 mM PMSF, 2 mM EDTA, and 10 mM dithriothreitol (DTT)). After 5 min, the above suspension was sonicated at 200 W for 15 min and then centrifuged at 30, 000 g for 15 min at 4 °C. The supernatant was carefully collected and 5× volume of chilled acetone containing 10 % (v/v) TCA was added, and the samples were mixed well and incubated overnight at −20 °C. After centrifugation at 30, 000 g for 15 min at 4 °C, the supernatant was discarded, and the precipitate was washed three times with chilled acetone. After air-drying, the precipitate was dissolved in lysis buffer containing 7 M urea, 2 M thiourea, 4 % NP40, and 20 mM Tris–HCl (pH 8.0-8.5). The suspension was sonicated at 200 W for 15 min and centrifuged at 30, 000 g for 15 min at 4 °C. The supernatant was collected and transferred to a new tube for further analysis.

### Polyacrylamide Gel electrophoresis and In-gel digestion

The proteins extracted from peanut samples were mixed with SDS loading buffer, and then denatured at 100 °C for 5 min. Proteins were separated on by 12 % polyacrylamide gel electrophoresis (SDS-PAGE) at 100 V. After electrophoresis, gels were stained with Coomassie Brilliant Blue R-250 (Pierce, USA) following the manufacturer’s instructions. Protein bands were sequentially and vertically cut into 10 slices, and each slice was placed in a separate Eppendorf tube. Each gel slice was cut into about 1-mm^3^ cubes, and was thoroughly destained using 50 mM ammonium bicarbonate containing 50 % acetonitrile (ACN) at 37 °C. Gel slices were incubated with 10 mM DTT containing 25 mM ammonium bicarbonate for 1 h at 60 °C to reduce disulfide bonds, and then alkylated with 55 mM iodoacetamide containing 25 mM ammonium bicarbonate for 45 min at room temperature in darkness. In-gel digestion was carried out by adding 0.01 μg/μL Trypsin Gold (Promega, USA) to the samples and incubating them at 37 °C overnight. After digestion, the peptides were further extracted twice using 0.1 % formic acid (FA) in 50 % ACN. The extracted peptides were dried and stored at −80 °C for LC-MS/MS analysis.

### LC-MS/MS experiments

The tryptic-digested peptide samples were redissolved in Buffer A (5 % ACN and 0.1 % FA), and centrifuged at 4 °C and 20, 000 g for 10 min to remove the insoluble material. The final concentration of peptide was about 0.5 μg/μl. Then, 10 μl of supernatant was loaded onto the nanoHPLC system (Shimadzu, Japan) according to the manufacturer’s instructions. The separation procedure was as follows. First, the peptides were loaded onto a 10-cm C18 trapping column (inner diameter, 75 μm; Waters, USA). In the separation process, samples were loaded at 8 μL/min for 4 min, and a 44-min gradient was then run at 300 nL/min starting with 2 to 35 % Buffer B (98 % ACN and 0.1 % FA), followed by a 2-min linear gradient to 80 % Buffer B, and maintenance at 80 % Buffer B for 4 min, and finally a return to 5 % Buffer B over a 1 min period. The peptides were subjected to nanoelectrospray ionization followed by tandem mass analysis.

The separated peptides were subjected to tandem mass spectrometry (MS/MS) in a LTQ Orbitrap Velos (Thermo, USA) coupled online to the HPLC system. Intact peptides were scanned in the Orbitrap at a resolution of 60, 000, and further selected for collision-induced dissociation (CID) operating with a normalized collision energy setting of 35 % [27]. The ion fragments were detected in the LTQ. A data-dependent procedure that alternated between one MS scan followed by ten MS/MS scans was applied, and the ten most intense ions above a threshold ion count of 5, 000 were detected with the following Dynamic Exclusion settings: repeat counts, 2; repeat duration, 30 s; and exclusion duration, 120 s. The applied electrospray voltage was 1.5 kV. Automatic gain control (AGC) was used to prevent overfilling of the ion trap and 1 × 10^4^ ions were accumulated in the ion trap to generate CID spectra. For the MS scans, the m/z scan range was 350 to 2, 000 Da.

### Data analysis and statistics

Raw data files obtained from the Orbitrap were converted into MGF files using Proteome Discoverer 1.2 (Thermo, USA). Protein annotation was performed using the Mascot search engine (Matrix Science, UK; version 2.3.02) against the peanut transcriptome database, which contained 72, 527 unigenes [10]. The parameters for protein identification are listed in Additional file 9: Table S9, and the false discovery rate (FDR) was set to 0.05 for both peptides and proteins.

Functional annotation of the proteins was performed using the Blast2 GO program against the non-redundant protein database at NCBI [28]. KEGG pathway analysis was performed by comparing the identified proteins with the KEGG database (http://www.genome.jp/kegg/). COG classification was carried out by comparing the identified proteins with the Cluster of Orthologous Groups database (http://www.ncbi.nlm.nih.gov/COG/).

### Availability of supporting data

The mass spectrometry of proteomics data have been deposited to the ProteomeXchange Consortium (http://proteomecentral.proteomexchange.org) [29] via the PRIDE partner repository with the dataset identifier PXD002579-81 and DOI 10.6019/PXD002579-81. The RNA-Seq data discussed in this publication have been deposited in NCBI’s Gene Expression Omnibus [30] and are accessible through GEO Series accession number GSE70972 (http://www.ncbi.nlm.nih.gov/geo/query/acc.cgi?acc=GSE70972).”

## Additional files

Additional file 1: Table S1. Specific proteins identified in S1 gynophores. (XLS 247 kb) Additional file 2: Table S2. Specific proteins identified in S2 gynophores. (XLS 147 kb) Additional file 3: Table S3. Specific proteins identified in S3 gynophores. (XLS 91 kb) Additional file 4: Table S4. Common proteins identified in S1, S2, and S3 gynophores. (XLSX 419 kb) Additional file 5: Table S5. Pathway analysis of identified proteins from S1, S2, and S3 gynophores. (XLS 778 kb) Additional file 6: Table S6. Transcriptome and functional analysis of specific proteins identified in S1 gynophores. (DOC 205 kb) Additional file 7: Table S7. Transcriptome and functional analysis of specific proteins identified in S2 gynophores. (DOC 94 kb) Additional file 8: Table S8. Transcriptome and functional analysis of specific proteins identified in S3 gynophores. (DOC 70 kb) Additional file 9: Table S9. Parameters of Mascot. (DOC 28 kb)

## Abbreviations

S1: Aerial grown gynophores
S2: Subterranean grown un-swelling gynophores
S3: Subterranean grown gynophores with early swelling pod
PRKM: Reads per kb per million reads
LC-MS/MS: Liquid chromatography-tandem mass spectrometry
GO: Gene Ontology
COG: Cluster of Orthologous Groups
KEGG: Kyoto Encyclopedia of Genes and Genomes
