# Supplementary material for: Proteomics analysis reveals differentially activated pathways that operate in peanut gynophores at different developmental stages
Source: BMC Plant Biol. 2015 Aug 4;15:188. doi: 10.1186/s12870-015-0582-6 (PMC4523997; doi:10.1186/s12870-015-0582-6)
Supplement: Additional file 6: Table S6. — Transcriptome and functional analysis of specific proteins identified in S1 gynophores. (DOC 205 kb) [file 12870_2015_582_MOESM6_ESM.doc]

Table S6 Transcriptome and functional analysis of specific proteins identified in aerial gynophores

| **Function annotation** | **Protein name** | **Accession no.** | **Protein Mass**  **(KDa)** | **Expression level (RPKM)** | | |
| --- | --- | --- | --- | --- | --- | --- |
|  |  |  |  | **S1** | **S2** | **S3** |
| **Cell Structure** | | | | | | |
|  | beta-D-xylosidase | Unigene63124 | 13561.04 | 42.35 | 16.61 | 17.37 |
|  | expansin | Unigene19425 | 21926.7 | 43.34 | 78.90 | 0.84 |
|  | polygalacturonase (pectinase) protein | Unigene70619 | 33460.94 | 97.03 | 46.50 | 31.07 |
|  | kinesin heavy chain-like protein | Unigene63270 | 16763.76 | 15.26 | 8.56 | 7.69 |
|  | Pectin methylesterase | Unigene70222 | 26254.8 | 146.01 | 91.03 | 48.06 |
|  | Pectin methylesterase | Unigene67076 | 20993.29 | 29.18 | 26.72 | 5.83 |
|  | Pectin methylesterase | Unigene15192 | 20662.78 | 51.51 | 16.62 | 6.95 |
|  | Pectin methylesterase | Unigene18179 | 41982.46 | 50.06 | 50.92 | 24.20 |
|  | polygalacturonase inhibiting protein | Unigene68584 | 21387.89 | 71.11 | 56.72 | 10.19 |
|  | villin 1 | Unigene70258 | 31708.85 | 142.74 | 73.82 | 28.29 |
|  | Villin 3 | Unigene7272 | 29917.13 | 79.04 | 31.42 | 16.84 |
| **Disease & Defense** | | | | | | |
|  | aspartic-type endopeptidase | Unigene70923 | 35985.53 | 69.45 | 37.91 | 14.13 |
|  | aspartyl protease protein | Unigene67059 | 22049.7 | 46.14 | 30.27 | 7.47 |
|  | Presequence protease 1 | Unigene12626 | 52521.97 | 36.73 | 15.55 | 18.32 |
|  | class III peroxidase | Unigene18372 | 34088.18 | 67.47 | 33.19 | 8.04 |
|  | LRR protein-related protein | Unigene65633 | 19449.98 | 8.13 | 3.09 | 0.52 |
|  | Putative L-ascorbate peroxidase chloroplastic | Unigene8506 | 19000.49 | 25.64 | 2.37 | 7.97 |
| **Energy** | | | | | | |
|  | alcohol dehydrogenase-like protein | Unigene68226 | 20515.9 | 79.65 | 20.67 | 29.47 |
|  | alcohol dehydrogenase-like protein | Unigene1324 | 9952.19 | 56.88 | 36.68 | 6.75 |
|  | ATP citrate lyase b-subunit | Unigene42705 | 7894.88 | 81.51 | 73.94 | 25.65 |
|  | NADH glutamate synthase precursor | Unigene45391 | 8948.37 | 60.74 | 20.96 | 6.91 |
|  | photosystem II protein D1 | Unigene64997 | 10693.33 | 2.56 | 0.29 | - |
|  | Pyruvate dehydrogenase E1 component subunit alpha mitochondrial | Unigene57644 | 10603.5 | 118.99 | 110.77 | 24.45 |
| **Metabolism** | | | | | | |
|  | acetylcholinesterase | Unigene27778 | 6519.16 | 30.29 | 12.20 | 8.13 |
|  | acid invertase | Unigene72190 | 57662.87 | 126.73 | 21.78 | 4.21 |
|  | aldose 1-epimerase protein | Unigene17218 | 34661.19 | 29.49 | 19.06 | 12.11 |
|  | alpha-galactosidase | Unigene61912 | 15552.51 | 93.73 | 38.61 | 13.23 |
|  | branched-chain alpha keto-acid dehydrogenase E1 | Unigene11052 | 26287.76 | 1.85 | 0.77 | 3.22 |
|  | enoyl-CoA hydratase/isomerase family protein | Unigene63339 | 12648.49 | 80.11 | 29.84 | 16.83 |
|  | GDSL-lipase 1 | Unigene39924 | 4927.42 | 49.57 | 14.57 | 1.94 |
|  | GDSL-motif lipase/hydrolase family protein | Unigene19153 | 39761.15 | 139.60 | 8.10 | 0.57 |
|  | geranylgeranyl hydrogenase | Unigene66761 | 21687.07 | 58.72 | 5.90 | 7.19 |
|  | glucosyltransferase [Ipomoea batatas] | Unigene62376 | 15742.81 | 53.80 | 2.08 | 0.32 |
|  | glucosyltransferase [Ipomoea batatas] | Unigene64302 | 18059.06 | 67.13 | 1.85 | - |
|  | Glycoside transferase six-hairpin subgroup [Medicago truncatula] | Unigene72099 | 51048.43 | 49.24 | 24.43 | 8.33 |
|  | GSDL-motif lipase | Unigene69514 | 28369.6 | 466.63 | 4.57 | 0.18 |
|  | methionine gamma-lyase | Unigene18665 | 11065.52 | 3.79 | 1.51 | 3.10 |
|  | Metal-dependent protein hydrolase family protein | Unigene17994 | 28163.06 | 1.67 | 0.96 | 0.36 |
|  | oxidoreductase | Unigene32237 | 6773.49 | 25.53 | 4.01 | 1.50 |
|  | 5-oxoprolinase/ hydrolase | Unigene4194 | 61615.29 | 19.47 | 19.32 | 21.11 |
|  | raffionse synthase 3 | Unigene19396 | 42331.48 | 37.42 | 18.25 | 10.93 |
|  | rhamnose synthase | Unigene71375 | 41544.49 | 32.69 | 21.58 | 7.30 |
|  | sucrose synthase | Unigene23894 | 6467.25 | 344.56 | 290.31 | 84.80 |
|  | UDP-galactose: SBMG-galactosyltransferase | Unigene28037 | 6397.32 | 117.10 | 83.15 | 21.83 |
|  | UDP-glucose:glucosyltransferase | Unigene16990 | 33415.02 | 15.80 | 6.26 | 4.52 |
| **Protein Destination & Storage** | | | | | | |
|  | serine protease-like protein | Unigene1742 | 19877.94 | 30.51 | 12.77 | 6.12 |
|  | DNAJ heat shock N-terminal domain-containing protein | Unigene69504 | 29786.75 | 22.64 | 11.06 | 12.10 |
|  | peptidyl-prolyl cis-trans isomerase cyclophilin-type family protein | Unigene68570 | 24647.06 | 19.25 | 0.86 | 1.20 |
|  | subtilisin proteinase | Unigene70206 | 26522.51 | 267.90 | 119.18 | 108.86 |
| **Protein Synthesis** | | | | | | |
|  | translation elongation factor EF-G | Unigene10836 | 77852.65 | 42.60 | 21.97 | 29.36 |
| **Secondary Metabolism** | | | | | | |
|  | anthocyanidin synthase 2 | Unigene71696 | 33056.16 | 105.49 | 35.11 | 1.14 |
|  | chalcone synthase | Unigene16603 | 22960.01 | 128.35 | 73.56 | 4.88 |
|  | cinnamoyl CoA reductase-like protein | Unigene68163 | 22784.82 | 221.16 | 178.55 | 34.34 |
|  | flavine-containing monoxygenase | Unigene68409 | 25844.73 | 14.77 | 6.55 | 8.97 |
|  | flavonoid 3-O-galactosyl transferase | Unigene67415 | 22595.33 | 154.98 | 0.73 | 0.90 |
|  | Phenylalanine ammonia-lyase 2 | Unigene68543 | 24605.74 | 46.92 | 58.69 | 17.46 |
|  | sedoheptulose-1 7-bisphosphatase | Unigene72218 | 42067.44 | 30.39 | 3.38 | 2.19 |
|  | serine decarboxylase | Unigene3735 | 19048.26 | 26.15 | 17.72 | 4.73 |
|  | starch-granule-bound R1 protein | Unigene65450 | 6594.41 | 73.45 | 53.55 | 42.01 |
|  | UDP-glucose:flavonoid 3-O-glucosyltransferase | Unigene59800 | 11859.2 | 262.57 | 2.35 | - |
| **Signal Transduction** | | | | | | |
|  | adenylyl cyclase associated protein | Unigene68552 | 19003.69 | 84.99 | 51.73 | 21.84 |
|  | leucine rich repeat protein | Unigene58583 | 12582.74 | 304.18 | 174.67 | 42.89 |
|  | leucine rich repeat protein | Unigene67728 | 11210.41 | 203.76 | 166.30 | 33.60 |
|  | leucine-rich repeat receptor-like protein kinase | Unigene68887 | 26263.38 | 17.79 | 15.73 | 7.53 |
|  | protein kinase | Unigene18824 | 16161.93 | 26.51 | 17.44 | 7.21 |
|  | protein phosphatase 2c | Unigene11209 | 54207.27 | 55.59 | 43.49 | 11.64 |
|  | SUB1; calcium ion binding | Unigene71124 | 29364.7 | 61.60 | 52.87 | 19.18 |
| **Transcription & Post-Transcription** | | | | | | |
|  | ascorbate oxidase precursor | Unigene71297 | 39464.2 | 231.28 | 84.83 | 19.67 |
|  | Iron-binding zinc finger CDGSH type domain-containing protein | Unigene66208 | 9024.79 | 53.42 | 18.80 | 27.82 |
|  | Zinc finger CCHC-type | Unigene17541 | 12755.03 | 49.29 | 13.36 | 18.40 |
|  | transcription factor style2.1 | Unigene58623 | 7679 | 119.58 | 62.33 | 7.32 |
| **Transporters** | | | | | | |
|  | Esterase/lipase domain-containing protein | Unigene61067 | 8813.57 | 83.40 | 59.42 | 8.61 |
|  | carbohydrate transporter/ sugar porter/ transporter | Unigene17294 | 46879.29 | 40.71 | 26.26 | 26.13 |
|  | lipase class 3 family protein | Unigene68480 | 26052.09 | 202.62 | 74.27 | 50.53 |
|  | multidrug/pheromone exporter MDR family ABC transporter | Unigene62432 | 16133.34 | 62.54 | 31.35 | 10.29 |
| **Unknown or Unclassified Function** | | | | | | |
|  | A_IG002N01.31 gene product [Arabidopsis thaliana] | Unigene59158 | 12699.66 | 8.58 | 3.23 | 1.13 |
|  | cephalopod | Unigene58454 | 13869.62 | 262.94 | 173.50 | 87.18 |
|  | endonuclease [Glycine max] | Unigene58710 | 13634.39 | 66.78 | 64.63 | 14.60 |
|  | F12K11.12 [Arabidopsis thaliana] | Unigene70368 | 30767.62 | 74.43 | 31.89 | 17.83 |
|  | Ricin B-related lectin | Unigene57143 | 9139.6 | 60.33 | 38.88 | 33.03 |
|  | Hexokinase-2 | Unigene71217 | 33458.95 | 27.01 | 16.73 | 7.48 |
|  | Hypothetical protein [Oryza sativa] | Unigene16241 | 11429.87 | 23.53 | 8.89 | 6.76 |
|  | light-harvesting complex I protein Lhca2 | Unigene17439 | 27699.17 | 27.09 | 2.26 | 6.34 |
|  | Porphobilinogen deaminase chloroplastic | Unigene18747 | 31971.6 | 62.36 | 24.66 | 17.86 |
|  | Porphobilinogen deaminase chloroplastic | Unigene13647 | 36850.85 | 21.14 | 6.07 | 9.87 |
|  | Porphobilinogen deaminase chloroplastic | Unigene71123 | 37578.82 | 19.84 | 6.90 | 7.91 |
|  | PTAC16 [Arabidopsis lyrata subsp. lyrata] | Unigene71480 | 29978.38 | 9.72 | 1.44 | 3.78 |
|  | root hair defective 3 | Unigene17398 | 49042.68 | 72.83 | 37.46 | 15.40 |
|  | RNA recognition motif (RRM)-containing protein | Unigene65351 | 17485.5 | 142.76 | 49.28 | 42.52 |
|  | RNA recognition motif (RRM)-containing protein | Unigene923 | 26958.92 | 45.80 | 19.34 | 20.65 |
|  | Tyrosinase | Unigene70498 | 30176.55 | 51.05 | 14.11 | 8.48 |
