# Supplementary material for: Proteomics analysis reveals differentially activated pathways that operate in peanut gynophores at different developmental stages
Source: BMC Plant Biol. 2015 Aug 4;15:188. doi: 10.1186/s12870-015-0582-6 (PMC4523997; doi:10.1186/s12870-015-0582-6)
Supplement: Additional file 7: Table S7. — Transcriptome and functional analysis of specific proteins identified in S2 gynophores. (DOC 94 kb) [file 12870_2015_582_MOESM7_ESM.doc]

**Table S7 Transcriptome and functional analysis of specific proteins identified in subterranean un-swelling gynophores**

| **Function category** | **Protein name** | **Accession no.** | **Protein Mass**  **(KDa)** | **Expression level (RPKM)** | | |
| --- | --- | --- | --- | --- | --- | --- |
|  |  |  |  | **S1** | **S2** | **S3** |
| **Cell Structure** | | | | | | |
|  | alpha-expansin 4 | Unigene68089 | 22483.92 | 97.48 | 96.19 | 27.89 |
|  | beta-D-glucosidase | Unigene17522 | 68378.24 | 36.64 | 51.66 | 39.67 |
|  | fasciclin-like AGP 14.8 protein | Unigene50173 | 8729.54 | 6.87 | 10.94 | 1.53 |
| **Disease & Defense** | | | | | | |
|  | class III peroxidase | Unigene2527 | 34895.52 | 27.73 | 136.27 | 17.73 |
|  | peroxidase 1 precursor | Unigene19499 | 27779.71 | 47.82 | 52.37 | 7.39 |
|  | peroxidase-like protein | Unigene51557 | 8999.51 | 7.62 | 107.25 | 14.23 |
| **Energy** | | | | | | |
|  | aldehyde dehydrogenase dimeric nadp preferring | Unigene44794 | 8487.36 | 119.38 | 327.77 | 198.15 |
|  | L-lactate dehydrogenase | Unigene17989 | 18928.94 | 18.63 | 36.01 | 12.21 |
|  | sugar-dependent lycerol-3-phosphate dehydrogenase | Unigene71221 | 38766.76 | 8.68 | 10.11 | 4.98 |
| **Intracellular Traffic** | | | | | | |
|  | delta-COP [Zea mays] | Unigene42225 | 8440.33 | 23.58 | 25.74 | 3.70 |
| **Metabolism** | | | | | | |
|  | acyl:coa ligase | Unigene71486 | 40972.23 | 9.21 | 43.37 | 15.49 |
|  | caffeic acid O-methyltransferase | Unigene56621 | 5866.94 | 2.58 | 8.47 | 1.67 |
|  | CXE carboxylesterase | Unigene22137 | 5173.75 | 27.07 | 130.50 | 5.41 |
|  | ADP-glucose pyrophosphorylase | Unigene12996 | 46079.87 | 45.87 | 50.05 | 14.76 |
|  | Os10g0155400 [Oryza sativa Japonica Group] | Unigene26514 | 6200.14 | 8.61 | 10.70 | 5.00 |
|  | phosphoenolpyruvate carboxykinase | Unigene65490 | 19680.55 | 8.79 | 54.31 | 37.16 |
| **Protein Destination & Storage** | | | | | | |
|  | cysteine protease | Unigene8297 | 27972.29 | 20.06 | 12.50 | 4.14 |
| **Secondary Metabolism** | | | | | | |
|  | anthocyanin-O-acyltransferase | Unigene47528 | 3775.9 | 65.00 | 98.92 | 31.69 |
|  | beta-amyrin synthase | Unigene65091 | 20050.76 | 205.18 | 190.22 | 86.87 |
|  | O-methyltransferase family 2; Dimerisation | Unigene61888 | 11749.6 | 105.27 | 201.32 | 56.35 |
|  | p-coumaryl-CoA 3'-hydroxylase | Unigene54676 | 11719.12 | 29.49 | 32.93 | 13.81 |
|  | phenylalanine ammonia-lyase | Unigene19719 | 5319.7 | 44.37 | 63.93 | 19.58 |
|  | phytase | Unigene49679 | 9826.72 | 44.48 | 54.94 | 16.58 |
| **Signal Transduction** | | | | | | |
|  | inositol phosphate kinase | Unigene19019 | 8774.38 | 7.34 | 10.92 | 6.65 |
| **Transcription & Post-Transcription** | | | | | | |
|  | auxin-responsive protein | Unigene69319 | 24396.91 | 18.13 | 62.79 | 15.16 |
|  | CND41 chloroplast nucleoid DNA binding protein | Unigene69340 | 27138.27 | 90.62 | 111.21 | 68.84 |
|  | S-like ribonuclease | Unigene69047 | 25521.98 | 8.02 | 18.02 | 6.43 |
| **Transporters** | | | | | | |
|  | Amino acid/polyamine transporter II | Unigene71843 | 32936.77 | 38.38 | 59.61 | 50.79 |
|  | MATE efflux family protein | Unigene70012 | 30582.44 | 14.49 | 23.83 | 6.10 |
|  | PDR-type ABC transporter 2 | Unigene71859 | 44479.44 | 48.56 | 188.56 | 91.96 |
|  | Sugar transporter ERD6-like 6 | Unigene8614 | 26855.24 | 32.31 | 41.87 | 10.28 |
| **Unknown or Unclassified Function** | | | | | | |
|  | 14-3-3 Protein [Glycine max] | Unigene64839 | 4418.18 | 4.89 | 8.64 | 1.95 |
|  | nodulin-like protein | Unigene68669 | 25648.57 | 34.84 | 80.86 | 46.76 |
|  | plastid-targeted protein 3 | Unigene16973 | 15604.16 | 22.22 | 51.54 | 19.25 |
|  | sieve element-occluding protein 3 | Unigene11826 | 24014.77 | 7.91 | 69.27 | 7.23 |
