# Supplementary material for: Proteomics analysis reveals differentially activated pathways that operate in peanut gynophores at different developmental stages
Source: BMC Plant Biol. 2015 Aug 4;15:188. doi: 10.1186/s12870-015-0582-6 (PMC4523997; doi:10.1186/s12870-015-0582-6)
Supplement: Additional file 8: Table S8. — Transcriptome and functional analysis of specific proteins identified in S3 gynophores. (DOC 70 kb) [file 12870_2015_582_MOESM8_ESM.doc]

**Table S8 Transcriptome and functional analysis of specific proteins identified in early swelling pods**

| **Function annotation** | **Protein name** | **Accession no.** | **Protein Mass**  **(KDa)** | **Expression level (RPKM)** | | |
| --- | --- | --- | --- | --- | --- | --- |
|  |  |  |  | **S1** | **S2** | **S3** |
| **Cell Growth/Division** | | | | | | |
|  | actin related protein | Unigene13778 | 20612.13 | 9.68 | 18.22 | 22.96 |
|  | actin-related protein 7 | Unigene68357 | 22205.06 | 5.73 | 7.91 | 31.79 |
| **Cell Structure** | | | | | | |
|  | microtubule-associated protein MAP65-1a | Unigene71866 | 45884.12 | 94.44 | 122.33 | 209.65 |
|  | Os02g0746000,Cullin-3 (CUL-3) | Unigene9263 | 33852.04 | 29.10 | 27.86 | 35.20 |
| **Disease & Defense** | | | | | | |
|  | oxidoreductase family protein | Unigene71316 | 33779.67 | 24.09 | 45.92 | 57.97 |
|  | coronatine-insensitive 1 | Unigene71597 | 45106.78 | 8.26 | 9.06 | 20.32 |
| **Energy** | | | | | | |
|  | OXP1,5-oxoprolinase | Unigene72458 | 71610.29 | 8.93 | 30.30 | 53.73 |
| **Metabolism** | | | | | | |
|  | lipoxygenase | Unigene19591 | 46288.5 | 13.36 | 36.54 | 34.43 |
|  | pyruvate dehydrogenase alpha subunit | Unigene17055 | 10945.49 | 10.32 | 20.85 | 23.11 |
|  | FAD-binding domain-containing protein | Unigene7949 | 43025.22 | 0.90 | 24.22 | 32.34 |
|  | anthranilate phosphoribosyltransferase | Unigene9663 | 32390.47 | 13.03 | 16.67 | 28.38 |
|  | starch branching enzyme I | Unigene69042 | 28579.65 | 22.91 | 35.48 | 41.83 |
|  | cinnamate 4-hydroxylase | Unigene62400 | 16410.58 | 103.69 | 267.22 | 81.22 |
|  | adenylosuccinate-AMP lyase | Unigene71360 | 41526.23 | 41.87 | 39.97 | 61.59 |
| **Protein Destination & Storage** | | | | | | |
|  | aspartic proteinase 1 | Unigene69126 | 26890.33 | 8.91 | 12.44 | 20.22 |
| **Secondary Metabolism** | | | | | | |
|  | homogentisate 1 2-dioxygenase | Unigene70146 | 25970.88 | 12.68 | 15.73 | 42.11 |
| **Signal Transduction** | | | | | | |
|  | SIT4 phosphatase-associated family protein | Unigene71567 | 41203.35 | 33.72 | 23.75 | 46.11 |
| **Transcription & Post-Transcription** | | | | | | |
|  | DNA binding/ zinc ion binding | Unigene13399 | 81592.96 | 27.68 | 20.60 | 47.89 |
|  | DNA binding protein binding | Unigene2730 | 32767.89 | 30.73 | 27.13 | 39.92 |
|  | nucleic acid binding protein | Unigene62821 | 15574.78 | 63.55 | 60.74 | 121.60 |
|  | binding | Unigene68861 | 27506.61 | 35.87 | 25.73 | 33.90 |
| **Unknown or Unclassified Function** | | | | | | |
|  | nodulins,MtN9 | Unigene58581 | 12026.11 | 9.19 | 20.70 | 200.54 |
|  | Os03g0596900[Oryza sativa] | Unigene66718 | 5459.64 | 18.65 | 27.55 | 47.35 |
|  | sister-chromatide cohesion protein 3 | Unigene19626 | 47471.87 | 29.44 | 22.55 | 39.32 |
|  | sieve element-occluding protein 3 | Unigene42923 | 8337.69 | 26.31 | 14.27 | 22.40 |
|  | Os02g0192100 [Oryza sativa Japonica Group] | Unigene56480 | 9655.6 | 19.50 | 23.78 | 29.73 |
