# Supplementary material for: Proteomics analysis reveals differentially activated pathways that operate in peanut gynophores at different developmental stages
Source: BMC Plant Biol. 2015 Aug 4;15:188. doi: 10.1186/s12870-015-0582-6 (PMC4523997; doi:10.1186/s12870-015-0582-6)
Supplement: Additional file 9: Table S9. — Parameters of Mascot. (DOC 28 kb) [file 12870_2015_582_MOESM9_ESM.doc]

**Table S9 Parameters of** Mascot

| **Item** | **Value** |
| --- | --- |
| Type of search | MS/MS Ion Search |
| Enzyme | Trypsin |
| Max Missed Cleavages | 1 |
| Mass Values | Monoisotopic |
| Peptide Mass Tolerance | 20 ppm |
| Fragment Mass Tolerance | 0.6 Da |
| Fixed modifications | Carbamidomethyl (C) |
| Variable modifications | Gln->pyro-Glu (N-term Q), Oxidation (M), Deamidated (NQ) |
| Instrument type Default | Default |
| Database | *Arachis hypogaea*(72527 sequences)^10^ |
